# Draft genome sequence of *Janibacter limosus* strain RCAM05316 isolated from Shulgan-Tash cave lime mud

**DOI:** 10.1128/MRA.00227-23

**Published:** 2023-10-31

**Authors:** Anna Sazanova, Andrey Belimov, Yuri Gogolev, Elizaveta Chirak, Alexey Afonin, Denis Karlov, Irina Kuznetsova, Polina Guro, Nina Tikhomirova, Vera Safronova

**Affiliations:** 1 All-Russia Research Institute for Agricultural Microbiology (ARRIAM), Saint Petersburg, Russia; 2 Kazan Institute of Biochemistry and Biophysics, FRC Kazan Scientific Center of RAS, Kazan, Russia; Indiana University, Bloomington, Bloomington, Indiana, USA

**Keywords:** *Janibacter*, genome, sequence, Shulgan-Tash

## Abstract

The Shulgan-Tash (Kapova) cave is a unique object for scientific research. In this article, we report the draft genome sequence of *Janibacter limosus* strain P1(28)-3 (RCAM05316) isolated from cave lime mud, Russia (53° 2′ 0″ N, 57° 3′ 0″ E). The sequence was obtained using Oxford Nanopore Technologies MinION.

## ANNOUNCEMENT

The Shulgan-Tash cave is located on the territory of the Burzyansky district of the Republic of Bashkortostan (Russia). This cave has specific conditions conducive to the formation of a consortium of chemoautotrophic microorganisms, including very low carbon content in the ground and high carbon dioxide content (1). It is expected that the metabolic interactions between members of such a consortium can be studied by comparative analysis of their whole genome sequences. Here, we present the complete genome of the strain *Janibacter limosus* RCAM05316, isolated from a lime mud sample taken in an extremely inaccessible part of the Shulgan-Tash cave—on the shore of the Dal`nee Verkhnee Lake. The sample was taken from the surface layer; aliquots of 10-fold dilutions of 1 g of the sample in sterile water were plated on a 10-fold diluted R2A agar medium. The strain *J. limosus* RCAM05316 formed white, flat, circular colonies after 9 days of incubation at 6°C. The culture was purified by streaking and stored at −80°C.

Genomic DNA was extracted and purified from a single colony using Monarch Genomic DNA Purification Kit (NEB, USA) according to the recommendation of the manufacturer.

Long-read whole-genome sequencing was performed using a MinION sequencer with the 9.4.1 rev. D flow cell (Oxford Nanopore, England). Isolated DNA without size selections was used with the SQK-LSK109 Ligation Sequencing Kit, and the EXP-NBD104 and EXP-NBD114 Native Barcoding Expansion kits were used to prepare the library according to the manufacturer’s instructions. The barcode 7 was used for the *J. limosus* RCAM05316 genome, 626.3 Mb, and 115,941 reads with an average length of 659,324 bp were generated for the strain. The reads were basecalled and demultiplexed, and the barcodes were trimmed using the guppy basecaller (v. 5.0.11, ONT, UK). The Flye assembler (v. 2.8) was used to assemble the Nanopore reads (2). The resulting assembly was corrected four times using Racon (v. 1.3.2) (3) with the following modifiers: (−m 8 −× −6 −g −8 −w 500), followed by a single polish using the medaka (v. 1.4.3) (4). The quality of the assemblies was evaluated with QUAST (v. 5.0.2) (5). The assembled genome of RCAM05316 consists of a single chromosome with a length 3,762,529 bp and an average GC content of 70.57%, genome coverage 189×. The circularity was reported by the Flye assembler and verified by mapping the long reads to the assembled fragments using Minimap2 (6), with the map-on mapping mode, and inspecting the coverage uniformity, no specific starting point was chosen for the assembled contigs. To correct frameshift errors in protein-coding regions, reference-based polishing was performed using protein sequences of the *J. limosus* NBRC 16128 type strain (GCF_001570985.1) using Proovframe software (7). Default parameters were used for all software unless otherwise specified. Search for genes in the assembled contigs was performed using the NCBI PGAP v. 5.3 (8). The 16S rRNA gene similarity between the strain RCAM05316 and the type strain *J. limosus* NBRC 16128 is 99.30%. We are aware of the limitations of genomes assembled solely from ONT data; therefore , the assembly cannot be considered as a high-quality complete genome.

## Data Availability

All data are available at GenBank under BioSample accession number SAMN23078441. The complete genome sequence was deposited under accession number CP087977 and the raw MinION data can be found under number SRR21050824.
